# Vitamin D status in diabetic Moroccan children and adolescents: a case-control study

**DOI:** 10.1900/RDS.2023.19.1

**Published:** 2023-03-31

**Authors:** Nisrine Bouichrat, Salma Benyakhef, Imane Assarrar, Najat Draoui, Youssef Lazreg, Naima Abda, Siham Rouf, Hanane Latrech

**Affiliations:** 1 Department of Endocrinology-Diabetology and Nutrition, Mohammed Vi University Hospital Center, Faculty of Medicine and Pharmacy, University of Mohammed First, Oujda, Morocco,; 2 Laboratory of Epidemiology, Clinical Research and Public Health, Faculty of Medicine and Pharmacy, University of Mohammed First, Oujda, Morocco.

**Keywords:** type 1 diabetes, vitamin D, utoimmunity, children and adolescents

## Abstract

**Background:**

Type 1 diabetes mellitus (T1DM) incidence is currently increasing worldwide, and different environmental players along with genetic predisposition, could be involved as powerful triggers of this disease onset. In this study, we aim to shed the light on the relationship between 25OHD deficiency and T1DM.

**Patients and methods:**

A case-control study was laid out to compare serum 25OHD level between T1DM patients and controls. A total of 147 T1DM patients aged under 19 years old were recruited from our Endocrinology-Diabetology and Nutrition department between October 2014 and December 2019. A total of 147 controls were randomly enlisted from clinical biochemistry laboratory of our center, and were carefully matched. The levels of 25OHD in the serum were determined in T1DM patients and nondiabetic controls.

**Results:**

Average serum 25OHD concentration was established in both groups; reaching 19,29 ±6,13 ng/ml in the control arm and 15,02 ± 6,48 ng/ml in the selected group with T1DM independently of the disease duration. However, the mean serum 25OHD concentration was not significantly different between the two T1DM subgroups according to diabetes duration below or above 5 years, and 25OHD concentration remained lower either in winter or summer months. A negative correlation was noticed between HbA1c and serum 25 OHD concentration in T1DM patients and was statistically significant (p<0,05).

**Conclusion:**

Key messages on the importance of vitamin D status, particularly in diabetic children and adolescents, should be spread widely in order to start a suitable vitamin supplementation, and establish guidelines regarding its timing at adequate recommended doses..

## 1. Introduction

Type 1 diabetes is commonly featured as a perplexing disorder involving a dynamic interaction between immunology; genetics and environment, leading to pre-defined beta-cell destruction with staging classification make over. The incidence of T1D has surprisingly increased during these last few decades by approximately 3-4% per year especially in low-incidence areas; related mainly to the impact of numerous environmental risk factors; including inter alia vitamin D. This hormone is not only necessary for phosphocalcic metabolism but is also involved in multiple immune-modulatory mechanisms [1]. In fact, It has been proved that almost all immune cells express the vitamin D receptor (VDR) under the command of 1,25(OH)2D upholding a plausible convenient immune activity of vitamin D supported by several animal studies [1]. In humans, different meta-analysis and international studies demonstrated a prominent 25OHD deficiency in subjects with T1DM [2], which might be conceptualized by different interconnected factors. The increase of T1D prevalence from northern areas to southern ones may be related to ecological matters or more precisely to the impact of sunlight exposure on vitamin D cutaneous synthesis [2]. Nevertheless, too many papers reported the potential impact of vitamin D deficiency on T1DM occurrence, progression and even on glycemic control. In this paper, we aimed to analyse the association between the serum 25OHD and T1DM in our local center, thus enrich the international data as it has never been investigated in our area and put the accent on the necessity of vitamin D supplementation.

## 2. Subjects and Methods

### 2.1 Study design and population

This is a case-control study laid out to compare serum 25OHD level between T1DM patients and controls, aged under 19 years old. All the patients admitted to Endocrinology-Diabetology and Nutrition department of Mohammed VI University Hospital in Oujda from October 2014 to December 2019, were enlisted. A suitable group of controls was randomly selected as outpatients of non-diabetic unit thanks to the valuable collaboration with the clinical biochemistry laboratory of our center.

They were carefully matched for gender, age, ethnicity, season of visit, and were living in the same geographical area, in order to provide a satisfying representative sample. The exclusion criteria of the study were: Age > 19 years old, consumption of Vitamin D or calcium or mineral supplementation within the previous 3 months, and co-morbidities or any other chronic diseases mainly influencing vitamin D metabolism.

This study was approved by the local Research Ethics Committee in line with the 1964 Declaration of Helsinki. A written consent was obtained from all participants in this study.

### 2.2 Data investigation

#### 2.2.1 Selecting patients with type 1 diabetes

A total of 147 diabetic individuals were identified, including 98 cases of new-onset diabetes and 49 participants already followed-up in our EndocrinologyDiabetology and Nutrition department. The total number of subjects with T1DM enrolled in our study was divided into two separate subgroups in consonance with diabetes duration, either less or more than 5 years including respectively 98 and 49 patients. Their HbA1c values have also been reported.

#### 2.2.2 Selecting controls

A total of 147 non-diabetic controls were randomly assigned. They have never been followed for any glucose metabolism disorder, and have never received any diabetes medication. Matching age, ethnicity, season of blood test and the same geographical locality have been necessarily taken into consideration while choosing the control group.

#### 2.2.3 Laboratory data

Serum 25-hydroxyvitamin D (25-OH D) levels have been efficiently identified in our population, based on Architect chemiluminescent immunoassay technology. This modern and easy approach, is designed as a delayed one-step microparticle immunoassay requiring programmed online pre-analysis with soft assay codes. Serum 25(OH)D2 and D3 can be both quantified by using antigen-antibody complexes (Polyclonal anti-vitamin D IgG antibody + microparticules) and biotinylated vitamin D anti-biotin IgG acridinium-marked compound. Nearly 5 ml blood were filled in appropriate laboratory sample tubes for all participants.

To date, there are no any available international guidelines defining properly reference levels, and figuring out vitamin D status. Nevertheless, most of the experts specify three separated sections according to 25-OHD measures: deficiency < 10 ng/ml, insufficiency: 10-30 ng/ml and sufficiency > 30 ng/ml [3].

### 2.3 Statistical analysis

Statistical Package for the Social Sciences, version 21 (IBM, Armonk, NY) was chosen as a software for data analysis. Descriptive statistics were mentioned for all variables as means, percentages and standard deviation. Data was normally distributed standing on Kolmogorov-Smirnov. Student t-test and Mann Whiteney tests were used to compare mean values of the assigned groups. Chi-square test and if necessary, Fisher’s exact test were performed to evaluate differences in proportions. For all statistical tests, a critical value of p < 0.05 indicated statistical significance.

## 3. Results

A total of 147 diabetic individuals were identified including 98 cases of new-onset diabetes and 49 participants already followed-up; besides 147 controls. Table 1 summarizes general data characteristics. There was a male predominance in both groups with a sex ratio of 1,07. The average age of participants in T1DM pool was 13,57 ± 4,08 years more represented by subjects aged above 12 years old (66,7%) with a mean diabetes duration around 3,56 ± 3,78 years; while the average age of healthy controls was 13,55 ± 3,36 years. The difference was not statistically significant (p=0,957).

**Table 1.** Features of T1DM study participants

		Controls (N=147) n (%)	Patients (N=147) n (%)	T1DM duration < 5 years (N=98)	T1DM duration > 5 years (N=49)
Gender	Boys	78 (53,1)	76 (51.7)	55	21
	Girls	69 (46,9)	71 (48.3)	43	28
Age (year)		13.55± 3.36	13.57± 4.08	12.73± 4.33	15.27± 2.91
Year	<12	49 (33.3)	49 (33.3)	41 (41.8)	8 (16.3)
	>12	98 (98)	98 (66.7)	57 (58.2)	41 (83.7)

Values are expresses as mean ± sd or n (%), Student t-test was used. A value of p<0.05 indicated significance.

Average serum 25OHD concentration was established in both groups; reaching 19,29 ±6,13 ng/ ml in the control arm and 15,02 ± 6,48 ng/ml in the selected group with T1DM independently of the disease duration. The difference was statistically significant (p=0,001) (Table 2).

**Table 2.** Assessement of Serum 25OHD level in T1DM cases and controls (ng/l)

	Controls (N=147)	Patients (N=147)	T1DM duration < 5 years (N=98)	T1DM duration > 5 years (N=49)
All	19.29±6.13	15.02±6.48	15.43±6.71	14.19±5.98
<12 years	21±5.01	15.74±7.23	16.47±7.63	12.02±2.66
>12 years	14.69±5.93	14.81±6.44	14.69±5.93	14.62±6.36

Values are expressed as mean ± sd, p<0,05 was statistically significant.

However, the mean serum 25OHD concentration was at 15,43±6,71 ng/ml when T1DM duration didn’t reach 5 years and 14,19±5,98 ng/ml when it exceeded 5 years (table 2). No significant statistical difference was noticed between the identified subgroups in T1DM participants (p=0,99).

Otherwise, vitamin D deficiency increased in females in both groups and was statistically significant (p=0,001), and increased more concretely with age in the T1DM patients.

**Figure 1.**
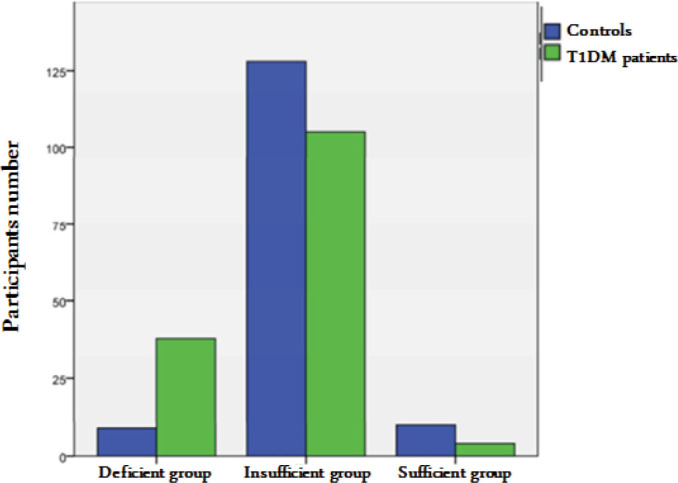
Vitamin D status in controls and T1DM

Vitamin D status was assessed in all of the studied groups (Figure 1). The highest proportion i.e., 25,85% (38/147) of deficient patients was noticed in the T1DM group, compared to the control group where the proportion was about 6,12% (9/147). However, the percentage of the insufficient group was higher (87,10% i.e.,128/147) in the control group, against 71,42% (105/147) in the cases.

Nonetheless, in T1DM subgroups, the proportion of the deficient group was 78,26% (36/49) in those with diabetes duration beyond 5 years and 69,47% (66/98) in those with a duration less than 5 years. The same trend was observed for the insufficient group with 27,36% (26/98) in the first subgroup (<5years) and 19,56% (9/49) in the second one (>5years). However, the difference was not statistically significant.

The seasonal variation of mean serum 25 OHD values in either controls or T1DM participants was elucidated in Table 3. Seasons were divided in two arms: Winter months (October to March) and summer months (April to September), in order to evaluate the fluctuation of vitamin D according to seasons all over the year, in controls and T1DM. In fact, 25 OHD levels of our patients were lowest than those of controls during winter months (p=0,0001) and summer ones as well (p=0,0031). The difference was then, statistically significant in both arms (Table 3).

**Table 3.** Seasonal variation of Vitamin D concentrations in T1DM

	T1DM patients	Controls	p-value
Winter months (83 patients, 83 controls)	14,62 ± 6,24	19,46 ± 5,88	0,0001
Summer months (64 patients, 64 patients)	15,53 ± 6,81	19,08 ± 6,49	0,0031

Winter months: (October to March), Summer months: (April to September).

**Figure 2.**
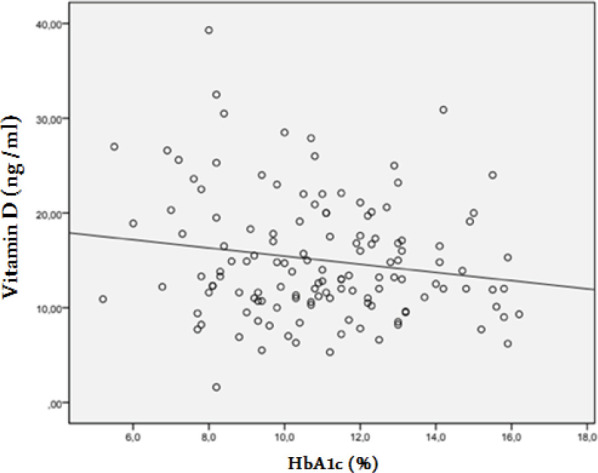
Linear correlation between vitamin D and HBA1c in T1DM group

In our study, 25 OHD values in T1DM group, showed significant negative correlation with HbA1c regardless of diabetes duration (Figure 2).

## 4. Discussion

Vitamin D deficiency is a major health issue in the world that goes well beyond disturbances in phosphocalcic homeostasis. In fact, immunomodulatory impact of vitamin D in T1D has widely been established either in animals or humans [4], relying on enhancing immune tolerance, counterbalancing autoimmune reaction, besides sustaining residual beta-cell function and decreasing disorder progression with an improved glycemic control [1,5]. Many experimental trials have undoubtedly illustrated the ability of vitamin D in preventing and modulating autoimmunity in nonobese diabetic (NOD) mice which have been adopted like the animal image of human T1D [6]. As a matter of fact, calcitriol constrains dendritic cells maturation and boosts their apoptosis [7], and hence avoids the immune response activation through intercepting their switch into antigen presenting cells [8].

Further studies pointed out the interactions between genes and environment. In MoBa study, higher concentrations of cord blood 25(OH)D were linked to a lower risk of T1D specifically among children who were considered as homozygous for the G allele at a particular VDR gene variant (variant rs11568820) [9]. Quite few single nucleotide polymorphisms (SNPs) exceeding 5000, have been determined for the VDR gene, and different studies have shed the light on the involvement of four of them in T1D susceptibility [10].

In the same way, the approach of Ponsonby et al. (2008) tends to enhance the impact of the environment in the association between VDR SNPs and T1DM [11]. But we can’t deny the plasticity of the immune system while accommodating a hereditary defect knowing that only few subjects with genetic vitamin D disorders may develop T1DM [12].

According to table 1, the average age of participants in our T1DM pool was 13,57 ± 4,08 years more represented by subjects aged above 12 years old (66,7%) comparatively to the report of the American investigators [13]. Vitamin D mean concentration was lower in T1DM participants compared to controls (table 2), and this difference was statistically significant; counting 71,42% of T1DM insufficient patients and 25,85% of deficient ones. This finding is in accordance with other recent published studies. Liu et al. reported in 2018 [14], vitamin D deficiency and insufficiency in 49,66% of 296 T1DM youths, with a lower mean serum 25OHD concentration in comparison to the control group (48.69 ± 15.26 nmol/L vs 57.93 ± 19.03 nmol/L). In the same line, the prevalence of vitamin D deficiency in a Swiss cross sectional case control study counting 129 T1DM subjects [15], was about 60,5% with a mean 25 OHD concentration of 28,8 nmol/l. It reached 43% in another Australian paper [16]; and approximated 25% in an Italian study [17] and 15% in an American one against 61% of insufficient patients [13]. The same results were observed in a Qatari case-control study involving 170 T1DM subjects with a vitamin D mean value of 15 ng/ml statistically different from healthy people vitamin D mean value (17 ng/ml; p=0,009) [18]; and in a Saudi study as well with vitamin D deficiency in 84% of T1DM children, against 59% of healthy ones [19].

In our study, no significant statistical difference was noticed between the identified subgroups in T1DM participants regarding diabetes duration (table 2) just like Mutlu et al. involving 120 cases with T1DM [20]. But, Ziaei-Kajbaf et al. determined that patients with more prolonged diabetes duration had lower 25OHD concentrations (p < 0.01) [21].

After all, the association between vitamin D deficiency and T1DM was not always reported. In fact, a study conducted in a solar rich environment in Florida enrolling 156 T1DM patients, didn’t enlighten any significant difference in vitamin D levels between the two study arms [22]. Moreover, an Egyptian study conducted by Azab et al. didn’t notice any significant low serum vitamin D level in diabetic participants (24.7 ± 5.6 vs 26.5 ± 4.8 ng/ml; P > 0.05) [23]. This has been also outlined by Ziaei-Kajbaf et al. in Iran [21] and Greer et al. in Australia [16]. Therefore, these observed variations might be spelled out by disparity in sun exposure as well as dietary intake or genetic predisposition from a wide point of view and geographical position. In fact, there is an obvious geographic variation regarding T1DM incidence; exhibited simply by the fact that a person who is living in Asia or Latin America is around 400 times less likely to develop the disorder than a child living in Nordic lands [24].

As a matter of fact, the seasonal variation in serum 25OHD concentrations is a well-documented fact, in many studies either in Finland (Agborsangaya et al. 2010; Holmlund-Suila et al. 2013) [25,26] or Sweden, (Klingberg et al, 2015) [27], Norway (Jorde et al, 2010) [28]. In our study (table 3), the impact of seasonal variation on vitamin D status was assessed in both controls and T1DM patients during summer and winter months, and it seemed that 25 OHD concentrations remained lower in T1DM compared to controls all over the seasons. Nevertheless, Liu et al. delineated that vitamin D level of T1DM patients was lower compared to controls only in summer period [14]. Otherwise, in a Kuwaiti cohort, no correlation has been established between seasons and vitamin D levels; regarding to clothing worn and life conditions mainly indoors just as the other countries in the Middle East area [29].

Many risk factors may be related to vitamin D deficiency such as sun exposure, clothing style, dietary intake of vitamin D, and certain pathologies (neoplasia, malabsorption...) [30]. In our series all patients are living in the same region and have the same sun exposure, similar Mediterranean diet and the same style of clothing. Also, our patients are not followed for any pathology that may lead to malabsorption.

As long as vitamin D is involved in T1DM pathogenesis, it would be interesting likewise to appreciate its effect on the glycemic control. In our study, there is a significant negative correlation between HbA1c and serum 25 OHD concentrations in T1DM patients (figure 2), going along the results of Svoren et al. in 2009 [13]; conducted in USA which pointed out vitamin D deficiency as one of the numerous factors involved in poor glycemic control. In Liu et al. report, HbA1c was associated to 25OHD concentrations only in established T1DM (diabetes duration>1 month) [14]. However, Tahereh Ziaei-Kajbaf in Iran [21] didn’t identify any significant correlation, as well as Daga et al. in northern India [31] and Mutlu [20].

Several interventional studies have been also conducted to highlight the importance of vitamin D supplementation in improving glycemic control [32,33]. However, there is up to date a harsh debate about an obvious effect of vitamin D supplementation in HbA1c level’s improvement. Vitamin D is believed to play a major protective role towards islet autoimmunity and beta-cell dysfunction as many papers have assessed the effect of vitamin D supplementation either during pregnancy or infancy on the decrease of T1DM occurrence. In fact, different studies noticed that the intake of cod liver oil which is rich of vitamin D and omega-3 PUFAs, all along before giving birth pursued till the first year, was related to a decreased risk of T1DM latterly [34,35]. Cadario et al. have published in 2018 an outstanding cohort study emphasizing the impact of mixing vitamin D to omega-3 PUFAs (150 mg of EPA and DHA/kg body weight) as part of a Mediterranean diet; in order to sustain residual beta-cell function and keep as long as possible, a partial clinical remission in subjects with newly diagnosed T1DM [36]. Indeed, there is an actual trial: Poseidon (Pilot Study of Omega-3 and Vitamin D in High-Dose in Type I Diabetic Patients) evaluating if one-year vitamin D supplementation by its self or added to omega-3 PUFAs is capable of maintaining beta-cell function and breaking the vicious circle of autoimmunity apart from the diabetes duration [37].

However, the suitable timing of starting the supplementation is crucial as displayed by Stene et al. in Norway [20]. Therefore, ruling out vitamin D supplementation from the seventh month till the end of the first year seems to reduce the risk of T1DM, in comparison to supplementation at birth till six months of life; reminding the lack of adaptive immune process during the first months of age; then vitamin D won’t be able to exert its immunomodulatory actions [38]. Nonetheless, recommendations about the best dose and the appropriate population to reduce the incidence of T1DM have not been yet determined [8].

The strengths of our study are mainly represented by the fact that our study is the first of its kind to be conducted in our country to compare vitamin status in diabetic and non-diabetic children and adolescents, and suggesting that vitamin D deficiency can be incriminated in T1DM pathogenesis. Our study sheds light on the importance of vitamin D screening and supplementation in order to attain glycemic control in patients with T1DM.

The limits of our study remain in the relatively limited number of our participants and the fact that it was monocentric. Larger multicentric studies are needed in order to assess an accurate prevalence of vitamin D deficiency in T1DM in our country and to study more explicitly the complicated causal relationship between them, taking into consideration environment and genetic background.

### 4.1 Conclusion

As long as the association between vitamin D deficiency and T1DM is witnessed in many studies conducted in different parts of the world, besides the involvement of vitamin D in immune-mediated cascade tending towards T1DM onset; it’s mandatory for every pratician and especially for endocrinologists to consider screening their T1D patients for vitamin D deficiency or insufficiency.

Public health message on the importance of vitamin D status, particularly in diabetic children and adolescents; should be spread widely in order to start a suitable supplementation which is reasonable, available and easily administered. But first and foremost, more longitudinal studies should be tailored in order to dissect carefully the causal relation between vitamin D in one hand and T1DM in the other hand; taking into consideration environment and genetic background in order to elaborate clear guidelines about vitamin D supplementation even in early stages of development, and thus promoting T1DM prevention.

## Abbreviations

T1DM: Type 1 diabetes mellitus

25OHD: 25-hydroxy vitamin D

T1D: Type 1 diabetes

VDR: vitamin D receptor

ISPAD: International society for pediatric and adolescent diabetes

HbA1c: Hemoglobin A1c

Omega-3 PUFAs: Omega-3 polyinsaturated fatty acids
